# Lack of association of colonic epithelium telomere length and oxidative DNA damage in Type 2 diabetes under good metabolic control

**DOI:** 10.1186/1472-6823-8-12

**Published:** 2008-10-10

**Authors:** Deepak Kejariwal, Karolina M Stepien, Tracy Smith, Hugh Kennedy, David A Hughes, Mike J Sampson

**Affiliations:** 1 Department of Gastroenterology, Norfolk and Norwich University Hospital Colney Lane, Norwich NR4 7UY, UK; 2 Elsie Bertram Diabetes Centre, Norfolk and Norwich University Hospital, Norwich NR4 7UA, UK; 3 Institute of Food Research, Norwich Research Park, Colney, Norwich NR4 7UA, UK

## Abstract

**Background:**

Telomeres are DNA repeat sequences necessary for DNA replication which shorten at cell division at a rate directly related to levels of oxidative stress. Critical telomere shortening predisposes to cell senescence and to epithelial malignancies. Type 2 diabetes is characterised by increased oxidative DNA damage, telomere attrition, and an increased risk of colonic malignancy. We hypothesised that the colonic mucosa in Type 2 diabetes would be characterised by increased DNA damage and telomere shortening.

**Methods:**

We examined telomere length (by flow fluorescent in situ hybridization) and oxidative DNA damage (flow cytometry of 8 – oxoguanosine) in the colonic mucosal cells of subjects with type 2 diabetes (n = 10; mean age 62.2 years, mean HbA1c 6.9%) and 22 matched control subjects. No colonic pathology was apparent in these subjects at routine gastrointestinal investigations.

**Results:**

Mean colonic epithelial telomere length in the diabetes group was not significantly different from controls (10.6 [3.6] vs. 12.1 [3.4] Molecular Equivalent of Soluble Fluorochrome Units [MESF]; *P *= 0.5). Levels of oxidative DNA damage were similar in both T2DM and control groups (2.6 [0.6] vs. 2.5 [0.6] Mean Fluorescent Intensity [MFI]; P = 0.7). There was no significant relationship between oxidative DNA damage and telomere length in either group (both p > 0.1).

**Conclusion:**

Colonic epithelium in Type 2 diabetes does not differ significantly from control colonic epithelium in oxidative DNA damage or telomere length. There is no evidence in this study for increased oxidative DNA damage or significant telomere attrition in colonic mucosa as a carcinogenic mechanism.

## Background

Type 2 diabetes mellitus (T2DM) is associated with a 40–60% increased risk of colorectal carcinoma [1,2]. The mechanisms underlying this association remain unclear. We have suggested recently [3] that one unexplored mechanism for this association is increased oxidative DNA damage and telomere attrition. Telomere-length abnormalities in epithelial cells occur very early in the development of many epithelial-derived tumours [4], including the transition from adenoma to carcinoma in colorectal epithelial cells [4,5].

Telomeres are tandem repeats of the hexanucleotide DNA sequence, TTAGGG at the end of eukaryotic chromosomes. Telomeres in somatic human cells shorten with every cell division and once shortened to a critical length become dysfunctional. Dysfunctional telomeres activate p53 to initiate cellular senescence or apoptosis to suppress tumorigenesis [6]. However, in the absence of p53, cells can escape the senescence checkpoint and continue to shorten their telomeres, resulting in entry into a phase of chromosomal instability characterized by chromosomal fusions and non-reciprocal translocations (NRTs) [7,8] commonly found in human epithelial carcinomas [9]). Loss of p53 function characterizes most human carcinomas and p53 mutations occur in 40–60% of colorectal adenocarcinomas [10,11].

Rates of telomere shortening, and therefore telomere length, are highly dependent on oxidatively-induced strand breaks in telomeric DNA and on cellular oxidant balance [12-15]. Telomeric DNA is particularly prone to oxidative damage at the GGG sequence, and it is probable that oxidatively induced single-and double strand DNA breaks in people with type 2 diabetes [16,17] would translate into accelerated telomere shortening and telomere dysfunction. We and others have shown that monocyte DNA from subjects with T2DM is characterised by increased susceptibility to oxidative damage and telomere shortening [16-18]. However we do not know if similar changes occur in tissues at risk of epithelial cancer development in T2DM. We hypothesized that patients with T2DM would demonstrate shorter telomeres in colonic epithelium compared with control subjects and this would be directly related to markers of oxidative DNA damage.

## Methods

All subjects gave written informed consent to participate in this study, which was approved by the local Research Ethics Committee. The patients were recruited from subjects attending our Gastroenterology Department for non urgent diagnostic endoscopy for clinical reasons. Up to six additional biopsies from the sigmoid colon were obtained for the study.

### Subjects and controls

Subjects were included if they were Caucasian non – smokers in an intentionally narrow age range of 50 – 70 years to limit confounding by age and other variables. Mean age in control group was 61.5 years and 62.2 among patients with T2DM. Male sex dominated with 15 in control group and 8 among patients with diabetes (Table 1). We excluded patients with abnormal lower GI endoscopy or patients with a previous history of inflammatory bowel disease, colon cancer or polyps. Subjects self medicating with any dietary vitamin or fish oil supplements were excluded. The T2DM patients were treated with metformin alone (n = 3), sulfonylureas alone (n = 1), metformin and sulfonylureas in combination (n = 3) or subcutaneous insulin (n = 2). Three of the 10 T2DM patients had background diabetic retinopathy (n = 2), nephropathy with macroproteinuria (n = 1) or peripheral neuropathy (n = 1). All subjects had a complete large bowel examination either by a colonoscopy or a flexible sigmoidoscopy and barium enema. The commonest indications for the evaluation of the large bowel were bleeding per rectum (n = 12 in control and n = 5 in type 2 diabetics) and change in bowel habit (n = 7 in control, n = 2 in diabetic subjects). Other indications for the test were family history of colorectal cancer (n = 3 in controls), anaemia (n = 2 in diabetics) and colorectal cancer screening (n = 1 in diabetics).

**Table 1:** Clinical features of type 2 diabetes and control groups

	Control subjects	Type 2 diabetes	P
N	22	10	
Age (years)	61.5 ± 5.5	62.2 ± 7.5	P = 0.76
M:F	15:7	8:2	
Diabetes duration (years)	-	6.5 (7.75)	
BMI (kg/m^2^)	26.6 ± 4.5	28.1 ± 6.9	P = 0.46
HbA1c (%)	-	6.9 ± 0.7	
Systolic blood pressure(mmHg)	145.8 ± 21.8	144.7 ± 16.9	P = 0.88
Diastolic blood pressure(mmHg)	89.6 ± 11.3	80.1 ± 9.3	P = 0.03
Statin use (n)	5	9	P = 0.0005
ACE inhibitors (n)	2	4	P = 0.05
Aspirin (n)	3	6	P = 0.01

Data are means ± SD or median (interquartile range).

BMI – body Mass Index

HbA1c – glycosylated hemoglobin

### Isolation of Cells

Six biopsies obtained from the sigmoid colon were immediately placed in Hanks Balanced Salt Solution (HBSS) (Sigma, UK) and transported on ice to the laboratory. Upon arrival (<60 min) biopsies were minced using a surgical scalpel and incubated with 3 mg collagenase (Roche Applied Science, UK) and 6 mg proteinase K (Sigma, UK) in 3 ml HBSS at 37°C for 1 – 1.5 h. Samples were resuspended every 15 min and once digested made up to 15 ml with HBSS. The suspension was then passed through a 70 um cell strainer (BD Falcon, UK) and centrifuged for 5 min 1400 rpm. The resulting pellet was resuspended in 1 ml cell freezing medium (Sigma UK) and divided equally between 2 cryovials. Cells were stored in liquid nitrogen prior to analysis.

### Telomere length

Telomere length of isolated mucosal cells was measured using a Dako Telomere PNA (Peptide Nucleic Acid) Kit/FITC (fluorescein isothiocyanate) for flow cytometry (Dako Cytometry, Ely, UK), as we have previously described [18], in combination with an anti-cytokeratin (CAM 5.2) (BD Biosciences, San Jose, CA) epithelial marker.

Thawed, cryopreserved cells were aliquoted into 2 tubes and resuspended in either 100 ul of hybridisation buffer containing the FITC/PNA probe or 100 ul of hybridisation buffer without probe. Both aliquots were then heated in a water bath at 82°C for 10 min, before being left in the dark at room temperature overnight to hybridise. After washing twice with wash buffer, 10 min at 40°C, 20 ul of PE (phycoerythrin) – labeled anti – CK (cytokeratin) was added to identify the epithelial population. Cells were then incubated at room temperature in the dark for 30 min before washing with PBS. Finally 125 ul of propidium iodide (PI) was added to each tube and incubated in the dark at 4°C for a minimum of 2 h prior to analysis. The mean telomere fluorescence of CK positive single cells was recorded by flow cytometry (Cytomics FC500MPL, Beckman Coulter) and calculated as the difference between the mean fluorescence of cells hybridised in the presence of the FITC-PNA probe and those hybridised in buffer. Telomere fluorescence data were then converted into molecular equivalent of soluble fluorochrome units (MESF) using Quantum premixed low level MESF beads (Bang Laboratories, Inc. Fishers, IN) which were run with each experiment.

### Oxidative DNA damage

Oxidative DNA damage in epithelial cells was assessed using a Biotrin OxyDNA test kit (Biotrin, Dublin, Ireland) in combination with the anti-CK epithelial marker. Immediately after cryopreserved cells were thawed and washed in phosphate buffered saline (PBS) they were fixed and permeablised using a Fix and Perm kit (Caltag Labs, Burlingame, CA). Oxidative DNA damage was determined using a FITC-labeled 8-oxoguanine probe as directed by manufacturer, as we have used previously [18]. However 30 min from the end of incubation with the FITC probe 20 ul of PE – labeled anti – cytokeratin (CAM 5.2) was added to label the epithelial population. Cells were then washed twice in PBSAA (PBS + 0.1% BSA + 0.02% azide) and the mean fluorescent intensity (MFI) of CK positive cells recorded by flow cytometry (Cytomics FC500MPL, Beckman Coulter).

### Data analysis and power calculations

Data are expressed as mean ± SD or as median (interquartile range) as appropriate. Differences between groups were analyzed by unpaired t test or Mann-Whitney U tests as appropriate and significance taken as P < 0.05. Differences between distributions of variables between groups were analyzed by Fishers exact text. No adequate previous data were available to allow sample size calculations. However, a total sample size of at least 40 between 2 groups would give 80% power at the 5% level to detect a one standard deviation difference between group means, which appears to be a biologically relevant difference [19,20].

## Results

### Clinical features

Clinical details of the two groups are shown (Table 1). The T2DM subjects were in good glycemic control (mean HbA1c 6.9%) after a median diabetes duration of 6.5 years.

### Telomere length by flow-fluorescent in situ hybridization and oxidative DNA damage

In the T2DM group, colonic epithelial cell (CK+) MESF was not significantly different than in control subjects (10.6 [3.6] vs. 12.1 [3.4]; p = 0.5). Similarly the MESF of total mucosal cell populations in the diabetic subjects were non significantly lower than in control subjects (8.2 [2.8] vs. 9.2 [2.8]; p = 0.6). Oxidative DNA damage (8-oxoguanine levels) in the colonic epithelial cells (CK+) was similar in both the diabetic and control subjects (2.6 [0.6] vs. 2.5 [0.6] MFI) (Table 2).

**Table 2:** Telomere length and oxidative DNA damage in subjects with or without type 2 diabetes

	Control subjects	Type 2 diabetes	P
n	22	10	
Telomere length			
CK+ cells	12.1 ± 3.4	10.6 ± 3.6	P = 0.27
All cells	9.2 ± 2.8	8.2 ± 2.8	P = 0. 38
Oxidative DNA damage			
CK+ cells	2.5 ± 0.6	2.6 ± 0.6	P = 0.76
All cells	2.2 ± 0.6	2.2 ± 0.6	P = 0.83

Data are means ± SD. Data as MESF for telomere length and MFI for 8-oxoguanine as a marker of oxidative DNA damage.

### Determinants of telomere length

There was no significant relationship between epithelial cell oxidative DNA damage and telomere length in either T2DM (r = +0.24, p = 0.5) or control groups (r = 0.32, p = 0.1). The relationship between age or BMI (Body Mass Index) and telomere length was not significant in either group.

## Discussion

The main findings in this study are that colonic epithelial cell telomere length and oxidative DNA damage were not significantly different in T2DM compared to controls, and that there was no significant relationship between oxidative DNA damage and telomere length in either group.

Colorectal cancer is characterized by sequential morphological changes of the colonic mucosa spanning the adenoma-carcinoma transition coupled with increasing genetic alterations. Telomere shortening, characterizes the adenoma-carcinoma transition, is apparent in the earliest detectable epithelial carcinoma stage [4,5], and in pre malignant states for colonic carcinomas [19,20].

Oxidative damage to DNA is widely thought to be a significant contributor to the age related development of major cancer [2,19,21]. We have recently shown an inverse association of telomere shortening and oxidative DNA damage [18] in circulating mononuclear cells in T2DM that could be due to increased oxidative DNA damage to monocyte precursors during cell division [18]. A number of other studies have also shown that systemic oxidative stress assessed by various biomarkers [23,24] is associated with shorter telomere lengths in peripheral blood leukocytes (PBL). These studies have used PBL telomere length as a systemic measure supported by the observation that telomere length is to a large extent conserved among different tissues. Telomere length could offer a link between oxidative stress and the predisposition to epithelial cancers in T2DM [3].

This is the first study measuring the telomere length and oxidative DNA damage in the colonic epithelium in type 2 diabetics, but we found no significant differences. The strength of this study is that the T2DM group was selected to limit confounding by variables that influence the risk of colorectal cancer such as race, age, smoking, polyps and inflammatory bowel disease. Moreover, the whole of the large bowel was imaged. This ensured that the measurements were truly from normal colonic mucosa. The main limitation of the study was small sample size in the diabetic patients. However, post hoc analysis suggests that this small study had 80% power to detect a difference of 2.66 MESF (0.7 SD) in the mean telomere length difference between the controls and T2DM at the 5% level.

Another possible confounding is the significantly higher use of aspirin, ACE and statins in the T2DM group. Available data suggest a neutral or protective effect of statins on telomere length or damage in vitro [25,26]. Aspirin has anti-inflammatory properties by inhibiting cyclooxygenase enzymes and substantially lower the risk of colon cancer [27]. ACE inhibitors have free radical scavenging properties and reduce oxidative stress [28,29]. It should also be stressed that the T2DM groups were in good glycemic control and glycemic control is an important risk factor for colorectal cancer [30]. The median duration of diabetes was 6.5 years. This may be another confounding factor as perhaps a longer duration of diabetes may be needed to demonstrate a toxic effect on the colonic epithelium.

## Conclusion

The present study therefore, found no data to support a substantially increased oxidative DNA damage or telomere attrition in the colonic mucosa in T2DM. This is an important area for investigation in view of growing interest in increased epithelial malignancy rates in T2DM.

## Abbreviations

T2DM: Type 2 diabetes mellitus; NRTS: non-reciprocal translocations; HBSS: Hanks Balanced Salt Solution; PNA: Peptide Nucleic Acid; MESF: molecular equivalent of soluble fluorochrome units; PBS: phosphate buffered saline; PE: phycoerythrin; FITC: fluorescein isothiocyanate; PBL: peripheral blood leucocytes.

## Competing interests

The authors declare that they have no competing interests.

## Authors' contributions

DK and KMS collected the data. DK analysed the data and drafted the manuscript. TS and DAH carried out the telomere length and oxidative DNA assays. DAH and HK helped in designing the study and drafting the manuscript. MJS conceived of the study, and participated in design and coordination and helped to draft the manuscript. All authors read and approved the final manuscript.

## Pre-publication history

The pre-publication history for this paper can be accessed here:


